# Glioma-Associated Antigen HEATR1 Induces Functional Cytotoxic T Lymphocytes in Patients with Glioma

**DOI:** 10.1155/2014/131494

**Published:** 2014-07-09

**Authors:** Zhe Bao Wu, Chao Qiu, An Li Zhang, Lin Cai, Shao Jian Lin, Yu Yao, Qi Sheng Tang, Ming Xu, Wei Hua, Yi Wei Chu, Ying Mao, Jian Hong Zhu, Jianqing Xu, Liang Fu Zhou

**Affiliations:** ^1^Department of Neurosurgery, Huashan Hospital, Fudan University, Shanghai 200040, China; ^2^Department of Neurosurgery, First Affiliated Hospital of Wenzhou Medical University, Wenzhou 325000, China; ^3^Department of Neurosurgery, Ruijin Hospital, Shanghai Jiao Tong University School of Medicine, Shanghai 200025, China; ^4^Key Laboratory of Medical Molecular Virology of Ministry of Education/Health at Shanghai Medical College, Shanghai Public Health Clinical Center and Institutes of Biomedical Sciences, Fudan University, Jinshan District, Shanghai 200040, China; ^5^Department of Immunology and Biotherapy Research Center, Shanghai Medical College, Fudan University, Shanghai 200040, China

## Abstract

A2B5+ glioblastoma (GBM) cells have glioma stem-like cell (GSC) properties that are crucial to chemotherapy resistance and GBM relapse. T-cell-based antigens derived from A2B5+ GBM cells provide important information for immunotherapy. Here, we show that HEAT repeat containing 1 (HEATR1) expression in GBM tissues was significantly higher than that in control brain tissues. Furthermore, HEATR1 expression in A2B5+ U87 cells was higher than that in A2B5−U87 cells (*P* = 0.016). Six peptides of HEATR1 presented by HLA-A∗02 were selected for testing of their ability to induce T-cell responses in patients with GBM. When peripheral blood mononuclear cells from healthy donors (*n* = 6) and patients with glioma (*n* = 33) were stimulated with the peptide mixture, eight patients with malignant gliomas had positive reactivity with a significantly increased number of responding T-cells. The peptides HEATR1_682–690_, HEATR1_1126–1134_, and HEATR1_757–765_ had high affinity for binding to HLA-A∗02:01 and a strong capacity to induce CTL response. CTLs against HEATR1 peptides were capable of recognizing and lysing GBM cells and GSCs. These data are the first to demonstrate that HEATR1 could induce specific CTL responses targeting both GBM cells and GSCs, implicating that HEATR1 peptide-based immunotherapy could be a novel promising strategy for treating patients with GBM.

## 1. Introduction

Human glioblastoma (GBM) accounts for approximately 60–70% of malignant gliomas, the most common and deadly brain tumors [1]. Despite improvements in standard therapies including surgery, radiation, and chemotherapy, the poor prognosis of patients with GBM has not been obviously improved. Immunotherapy represents a promising treatment designed to reshape the immune system to specifically eradicate malignant cells. The effort of T-cell-mediated immunotherapy to selectively kill remnant glioma cells that could not be completely removed using microsurgery has been highlighted [2–4].

Glioma stem-like cells (GSCs) may be capable of initiating tumor growth [5, 6] and are likely to be responsible for the malignant behavior of tumors because of their acquired resistance to chemotherapy, radiotherapy, and immunotherapy induced by glioma-associated antigens, which results in the ineffectiveness of existing conventional therapies [7–9]. Thus, GSCs could be a novel target for cancer therapy, including immunotherapy. Our recent study findings indicated that glioma stem-like cell-associated antigens (SAAs) from CD133+ GSCs bear highly immunogenic antigens and induce significant responses from cytotoxic T lymphocytes (CTLs) [10]. Several other studies have tried immunotherapy targeting GSCs [11–16].

A2B5 is considered a marker for immature glial-committed progenitors that are permanently generated in the subventricular zone. Glial progenitor cells are defined as cells that give rise to glial cell types such as astrocytes and oligodendrocytes. In GBM tissues, A2B5+ cells include A2B5+/CD133+ and A2B5+/CD133− cells. Furthermore, A2B5+ cells from human GBM have cancer stem-like cell properties that are crucial for the initiation and maintenance of GBM [17, 18]. Thus, A2B5+ GBM cells could be an ideal target for GBM immunotherapy. Our recent study found that vaccination with A2B5+ GL261 cell lysate-pulsed dendritic cells had a preventive effect for mouse glioma [19]. However, T-cell epitopes derived from A2B5+ GBM progenitor cells for immunotherapy have not been reported.

To identify novel genes selectively overexpressed in A2B5+ GBM as the target for T-cell mediated immunotherapy, we sequenced the mRNA profile of A2B5+ GBM cells from U87 cell lines using fluorescence-activated cell sorting (FACS) by Solexa sequencing (data not shown) and identified that the HEAT repeat containing 1 (HEATR1) gene (gene ID: 55127) was overexpressed in A2B5+ GBM cells. Recently, Bleakley et al. reported that HEATR1 was highly expressed in testis and ovary than in other tissues including liver, colon, small intestine, lung, brain, and heart [20]. Identification of epitope derived from HEATR1 is likely to provide alternative candidates for the design of antitumor vaccine with high efficacy in the future.

In the present study, we confirm the selective HEATR1 overexpression in A2B5+ GBM cells and in the vast majority of GBM. In addition, we identify several HEATR1-derived T-cell epitopes in tumor carrier patients. Our results emphasize the suitability of this protein for T-cell-based immunotherapy in patients with GBM.

## 2. Materials and Methods

### 2.1. Ethics Statement

The study protocol was approved by the Local Independent Ethics Committee at Huashan Hospital, Fudan University. Some samples in this study were used in our previous reports [21, 22]. Written informed consent was obtained from each donor of the samples used in our research.

### 2.2. Cell Lines

Human GBM cell lines U87, A172, and SHG66 were used in this study. SHG66 came from a 47-year-old man with a right parietal glioblastoma (World Health Organization grade IV) [10]. U87 and A172 cells were purchased from the cell bank of the Shanghai Branch of Chinese Academy of Sciences. A172 cells did not express HLA-A∗02:01 [23, 24], while the other two GBM cell lines expressed HLA-A∗02:01 according to flow cytometry [25, 26]. The HLA-A∗02:01, expressing human tumor cells T2 (deficient in TAP1 and TAP2 transporters), and BB7.2 hybridoma, producing anti-HLA-A∗02 monoclonal antibody (mAb), were purchased from American Type Culture Collection (USA). All cell lines were cultured in Dulbecco's modified eagle medium (DMEM; Gibco, Grand Island, NY, USA) supplemented with 10% fetal bovine serum (FBS; Hyclone, Logan, UT, USA) and 100 U/mL penicillin/streptomycin (Gibco) and maintained in a humidified atmosphere with 5% CO_2_ at 37°C.

The GSC lines (U87, A172, and SHG66) were established and characterized as described previously [10, 13]. Short-term tumor spheres of the GBM cell lines were cultured in serum-free medium (SFM) consisting of DMEM/F12 (Invitrogen) supplemented with 20 ng/mL recombinant human basic fibroblast growth factor (bFGF; Chemicon), 20 ng/mL recombinant human epidermal growth factor (EGF; Chemicon), and B27 (Invitrogen). The GSC tumor spheres exhibited stem cell-like characteristics [10, 15].

### 2.3. Patients

A total of 22 frozen GBM tumor tissues were obtained from the Department of Neurosurgery, Huashan Hospital, to analyze the expression level of HEATR1 mRNA. Additionally, eight control brain tissue samples were obtained from adjacent brain tissues of patients with traumatic brain injury who suffered contusion and laceration. In addition, 10 GBM formalin-fixed, paraffin-embedded (FFPE) tissue sections and 10 normal brain tissues were analyzed by IHC.

Peripheral blood mononuclear cells (PBMCs) were isolated by Ficoll/Paque (Biochrom, Berlin, Germany) density gradient centrifugation of heparinized blood obtained from healthy donors (*n* = 6) and patients (benign tumors, 5; grade 2 astrocytoma, 7; grade 3 anaplastic glioma, 10; glioblastoma, 16). The patients' clinical characteristics are listed in Table 1.

### 2.4. FACS with A2B5

The U87 cells were resuspended at a density of 1 × 10^5^ cells/mL in SFM consisting of DMEM/F12 (Invitrogen) supplemented with 20 ng/mL recombinant human bFGF, 20 ng/mL recombinant human EGF, and B27. U87 cells were cultured for 2 weeks. A2B5-PE antibody (Miltenyi Biotec) was used in this study for FACS. Cell sorting was performed on a BD FACSVantage Cell Sorter (BD Biosciences) according to the manufacturer's instructions.

### 2.5. Real-Time Reverse Transcription-Polymerase Chain Reaction (RT-PCR) of HEATR1 Expression

Total RNA was extracted from GBM and control brain tissues or from the GBM cell lines using Trizol reagent (Invitrogen) according to the manufacturer's instructions. First-strand cDNAs were synthesized using a High-Capacity cDNA Archive Kit. Each cDNA (2 *μ*L) was amplified in a SYBR Green Real-time PCR Master Mix (final volume, 20 *μ*L) and loaded on an Applied Biosystems 7900 Real-time PCR Detection System (Applied Biosystems, Foster City, CA, USA). Thermal cycling conditions for quantitative RT-PCR (qRT-PCR) were as follows: the first step, 95°C for 10 min and the ensuing 40 cycles, 95°C for 15 s, 60°C for 60 s, and 72°C for 30 s. The qRT-PCR primers used were as follows: HEATR1 (forward) 5′-TCCTTTTTGATACCCAGCATTTTAT-3′ and HEATR1 (reverse) 5′-TGATCCACCAGAGGCATCATC-3′; actin (forward) 5′-CCCTGGCACCCAGCAC-3′ and actin (reverse) 5′-GCCGATCCACACGGAGTAC-3′. All samples were analyzed in triplicate. To validate that the efficiencies of the target gene amplification and *β-ACTIN* amplification were approximately equal, we plotted standard curves of log input amount versus ΔCT (CT_target_–CT_control_) for every gene and all the slopes of the plot <0.1. The ΔΔCT method recommended by the manufacturer was used to compare the relative expression levels between samples.

### 2.6. IHC Analysis

Human GBM FFPE tissue sections were provided and IHC stained with HEATR1-specific antibody made against COOH-terminal peptide of human HEATR1 (Sigma-Aldrich) using a DakoCytomation EnVision+ System-HRP (DAB) detection kit. Briefly, 5 *μ*m tissue sections were dehydrated and subjected to peroxidase blocking. HEATR1 antibody was added at a dilution of 1 : 20 and incubated at room temperature for 30 min on the Dako Autostainer using the DakoCytomation EnVision+ System-HRP (DAB) detection kit. The slides were counterstained with hematoxylin. The stained slides were observed under a microscope and images were acquired. Cytoplasm staining was considered positive. To evaluate HEATR1 expression, 10 high-power fields (400x) within the tumor showing cytoplasm staining were selected. IHC signals were visually quantified by L.F. Sempere using a quick score system combining staining intensity and positive cell percentage (staining intensity: 0 = negative, 1 = weak, 2 = intermediate, and 3 = strong; percentage: 0 = 0%, 1 = <25%, 2 = ≥25%, and 3 = ≥50%). All of the IHC stained sections were evaluated by two senior neuropathologists blinded to the clinical parameters.

### 2.7. Peptide HLA-A∗02:01 Binding Affinity

The binding activity of selected peptides to the HLA-A∗02 molecule was determined semiquantitatively by measuring peptide-induced expression of HLA-A∗02:01 on T2 cells using flow cytometry. The T2 cells were incubated for 4 h with the candidate peptides, respectively, at a concentration of 20 *μ*g/mL in SFM. After being washed with phosphate buffered saline-fetal calf serum (PBS-FCS), the T2 cells were incubated with supernatant containing murine mAb against HLA-A∗02:01 derived from BB7.2 cells for 30 min at 4°C. The T2 cells were washed twice with PBS-FCS and stained with 5 *μ*g/mL diluted fluorescein isothiocyanate-conjugated immunoglobulin G which reacts to mouse immunoglobulin for 30 min. The cells were then rinsed three times with PBS-FCS and analyzed using a FACSAria flow cytometer. The percent mean fluorescence index (% MFI) increase of HLA-A∗02:01 molecules was calculated as follows: % MFI increase = [(MFI with peptide − MFI without peptide)]/(MFI without peptide) × 100 [27].

### 2.8. Interferon-*γ*- (IFN-*γ*-) Based Enzyme-Linked Immunosorbent Spot (ELISpot) Assay

A human IFN-*γ* ELISpot kit (552138; BD Pharmingen, CA) was used to quantify the CTL response in PBMCs. Several 96-well plates were coated with purified anti-human IFN-*γ* monoclonal antibodies at the concentration of 5 *μ*g/mL at 100 *μ*L/well and incubated at 4°C overnight and then washed once with 200 *μ*L/well of RPMI-1640 containing 10% FBS and 1% penicillin-streptomycin-L-glutamine (R10) and blocked with 200 *μ*L/well R10 for 2 h at room temperature. PBMCs were then washed twice with R10 and resuspended in R10 complete culture medium. After being counted, the cells were then adjusted to the concentration of 1 × 10^6^ cells/mL and plated onto a 96-well ELISpot plate at 50 *μ*L/well (5 × 10^4^ cells/well) with the addition of 50 *μ*L of the peptide. The final concentration of each peptide was 5 *μ*g/mL. The 96-well ELISpot plates were incubated for about 20 h at 37°C in 5% CO_2_. After incubation, the ELISpot plates were developed according to the kit instructions. Finally, the plates were air-dried and the resulting spots were counted with ChampSpot IV Bioreader (Beijing SAGE Creation Science, Beijing, China). Peptide-specific IFN-*γ* ELISpot responses were considered positive only when the number of spots was twofold greater than the control peptide stimulation and there were >50 spots per 1 × 10^6^ PBMCs [28, 29].

### 2.9. Cytotoxicity Assay by Measuring Lactate Dehydrogenase (LDH) Activity

CytoTox 96 Nonradioactive Cytotoxicity Assay (Cat. number G1780, Promega) was used to determine the cell-mediated cytotoxicity [27, 30]. U87, SHG66, and A172 cells serving as target cells (1 × 10^5^) were loaded with 4 *μ*g/mL peptide for 2 h at 37°C and 5% CO_2_. Effector PBMCs (1 × 10^6^) were added to peptide-loaded or blank target cells and cultured for additional 4 h at 37°C and 5% CO_2_. To measure the LDH activity, 50 *μ*L of the reconstituted substrate mix was added to 50 *μ*L of the culture supernatant and incubated at room temperature protected from light for 30 min. A total of 50 *μ*L of the stop solution was added to each well of the plate. The concentrations of the colorimetric product were recorded as absorbance at 490 nm by a spectrometer [27].

### 2.10. Statistical Analysis

All statistical analyses were carried out using the SPSS 16.0 statistical software package. Continuous variables are expressed as mean ± SEM. Statistical differences between the two groups were evaluated using the unpaired Student's *t*-test. The correlation between ELISpot response and glioma grades was evaluated using the *χ* ^2^ test. *P* values < 0.05 were considered statistically significant (two-tailed test).

## 3. Results

### 3.1. HEATR1 Overexpression in GBM and A2B5+ GBM Cells

**Figure 1:**
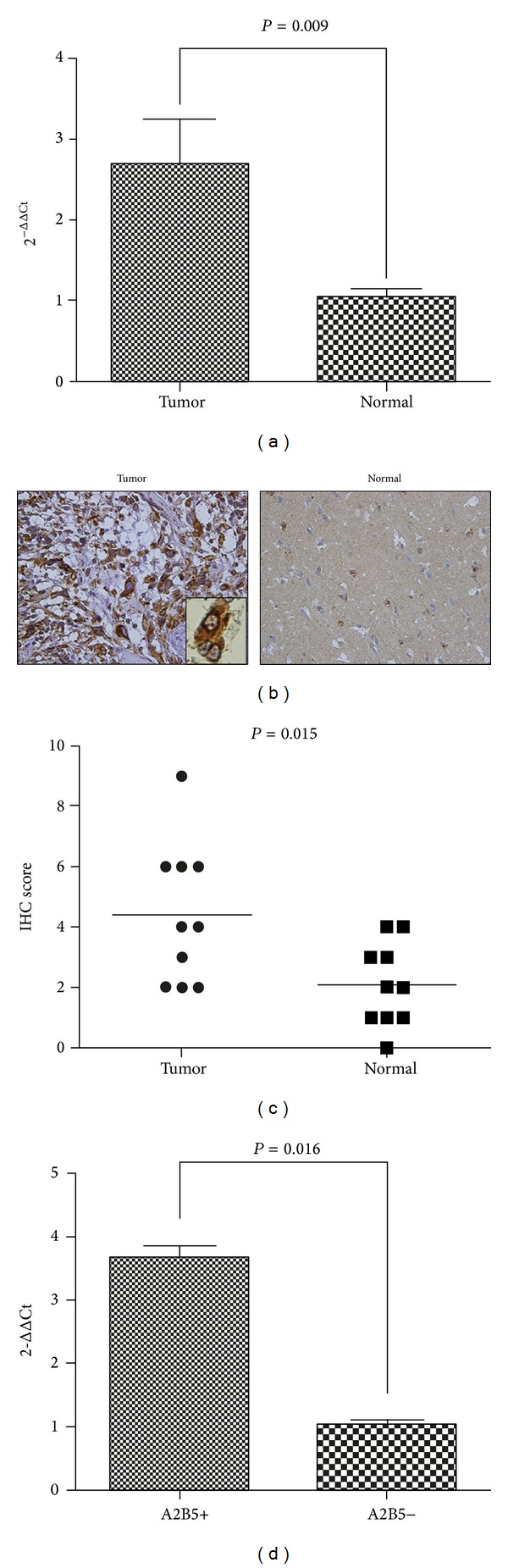
HEATR1 was overexpressed in GBM and in A2B5+GSCs. (a) qRT-PCR was performed to analyze the differential expression between GBM tissues (*n* = 22) and controlled brain tissues (*n* = 8). (b)-(c) IHC was performed in FFPE tissue sections of 10 primary GBM tissues (left, ×400) and 10 normal brain tissues (right, ×400). GBM tissues had higher staining score of HEATR1 protein than normal brain tissues (*P* = 0.015). (d) qRT-PCR was performed to analyze the differential expression between A2B5+U87 cells and A2B5−U87 cells (*P* = 0.0016).

First, we investigated whether HEATR1 was overexpressed in GBM cells. We investigated the expression profile of HEATR1 mRNA in 22 primary GBM tissues and eight control brain tissues using quantitative RT-PCR. As shown in Figure 1(a), the expression of HEATR1 mRNA in GBM tissues was higher than that in control brain tissues (*P* < 0.01). In addition, IHC was initially performed in FFPE tissue sections of primary GBM (*n* = 10) and normal brain tissues (*n* = 10). As shown in Figure 1(b), HEATR1 protein was mainly localized in the tumor cell cytoplasm and nuclei. The average IHC score of HEATR1 expression in GBM and normal brain tissues was 4.4 ± 0.7 and 2.1 ± 0.4, respectively. GBM tissues had higher expressions of HEATR1 protein than normal brain tissues (Figure 1(c), *P* = 0.015). However, the expression level of HEATR1 proteins did not appear to be correlated with glioma grade (data not shown).

Next, we investigated whether HEATR1 expression in A2B5+GBM cells was higher than that in A2B5−GBM cells. Our previous study showed that U87 cells cultured in SFM for 2 weeks had stem-like features [10]. Furthermore, those A2B5+ U87 cells were double-positive for CD133 and nestin or vimentin (Supplementary Figures 1, 2, and 3, resp., in the Supplementary Material available online at http://dx.doi.org/10.1155/2014/131494). Prior to sorting, the percentage of A2B5+ cells accounted for 6.5%. HEATR1 mRNA in sorted A2B5+ U87 cells was significantly higher than that in A2B5−U87 cells quantified by qRT-PCR (*P* = 0.016, Figure 1(d)).

### 3.2. Prediction of Candidate HLA-A∗02-Binding Peptides Derived from HEATR1

Due to HEATR1 overexpression in GBM, we sought to determine whether HEATR1-derived epitopes that could be presented by antigen process machinery and induce the CTL response in patients with GBM. Since HLA-A∗02:01 is expressed by 30–40% of Asians as the most common subtype of HLA-I class [31, 32], epitopes potentially binding to HLA-A∗02:01 were generated using the HLA Peptide Binding Predictions Program (http://www-bimas.cit.nih.gov/molbio/hla_bind/) of the Bioinformatics and Molecular Analysis Section [12]. Six peptides with binding scores >1000 were selected as the candidate epitope peptides (Table 2). Peptides including HEATR1_2003–2011_ (2003–2011, FLFDTQHFI), HEATR1_1126–1134_ (1126–1134, KLLRMLFDL), HEATR1_2102–2110_ (2102–2110, LLPESIPFL), HEATR1_1411–1419_ (1411–1419, FLWILLILL), HEATR1_682–690_ (682–670, KMVEDLISV), and HEATR1_757–765_ (757–765, LMLDRGIPV) were synthesized by GL Biochem (Shanghai) Ltd. with >95% purity as indicated by analytic high-performance liquid chromatography and mass spectrometric analysis. The negative control peptides (CFLPVFLAQPPSGQR) were also synthesized.

### 3.3. Affinity of Candidate Epitope Peptides for HLA-A∗02 Molecule

**Figure 2:**
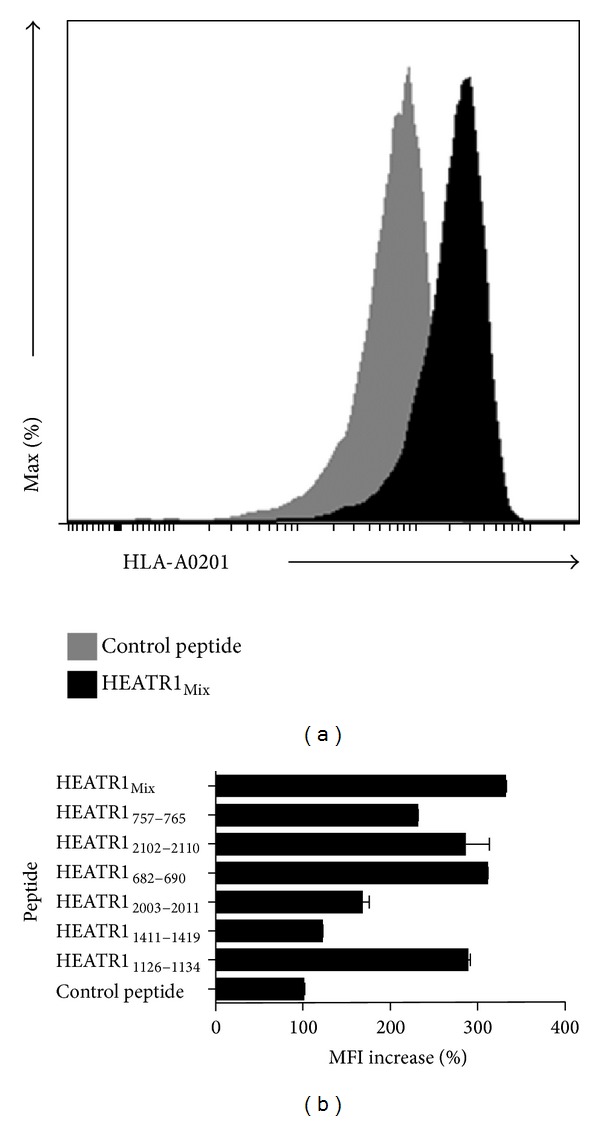
HLA-A02 binding affinity of six candidate peptides. (a) Flow cytometry results of HEATR1_mix_. (b) The binding activity of selected peptides to HLA-A∗02 molecule was determined semiquantitatively by measuring peptide-induced expression of HLA-A∗02 on T2 cells with flow cytometry. Data from three independent experiments were expressed as the mean ± SE. Unrelated 15-mer peptides were considered as control peptide.

The T2-cell-peptide binding test was used to evaluate the binding affinity of these candidate epitope peptides for HLA-A∗02 with flow cytometry* in vitro* (Figure 2(a)). As shown in Figure 2(b), HEATR1_682–690_ had the highest affinity for HLA-A∗02:01 and the percentage of MFI increase was 308.5 ± 4.8%. The percentages of MFI increase of HEATR1_2102–2110_, HEATR1_1126–1134_, and HEATR1_757–765_ were 285.2 ± 49.2%, 287.2 ± 7.7%, and 228.7 ± 5.4%, respectively. HEATR1_2003–2011_ was a lower affinity peptide, while HEATR1_1411–1419_ had the lowest affinity for binding to HLA-A∗02.

### 3.4. HEATR1-Derived Peptides Induced CTL Responses

**Figure 3:**
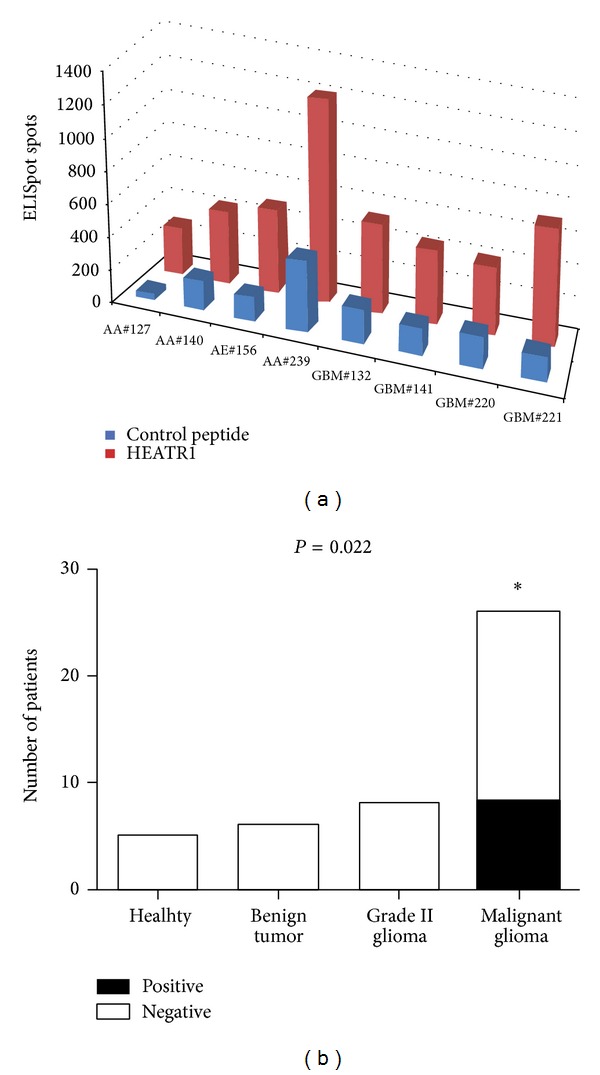
Six epitope peptides derived from the HEATR1 induce the IFN-*γ* response. (a) ELISpot result of 8 malignant gliomas with positive reactivity. The number of IFN-*γ* formingspots was calculated per 1 × 10^6^ PBMCs. (b) The positive reactivity among 6 healthy donors and 38 patients only occurred in 8 malignant gliomas (*P* = 0.022). GBM: glioblastoma multiforme; AA: anaplastic astrocytoma; AE: anaplastic ependymoma. This is a representative experiment from two independent experiments. No peptide stimulation was negative control. Correlation between ELISpot response and glioma grades was evaluated using a *χ* ^2^ test.

In the next set of experiments, we tested whether those candidate peptides are epitopes that can be recognized by the host immune system* in vivo*. PBMCs from glioma carriers were incubated with those six mixed peptides and the IFN-*γ* secretion was tested by the ELISpot. As shown in Table 1, we found that eight patients (anaplastic astrocytomas/ependymoma in four and glioblastoma in four) had positive reactivity with a significant increase of ELISpot-detected spots (Figure 3(a)). The frequency of positive reactivity in malignant gliomas accounts for about 31%. In this study, those positive responses were only observed in the malignant glioma, indicating that those epitopes could be considered specific for malignant gliomas and significantly higher than healthy donors and low-grade glioma carriers (Figure 3(b), *P* = 0.022). In addition, three of eight patients with positive reactivity were non-HLA-A∗02 (Supplementary Table 1), indicating that these peptides might not be exclusively presented by HLA-A∗02.

**Figure 4:**
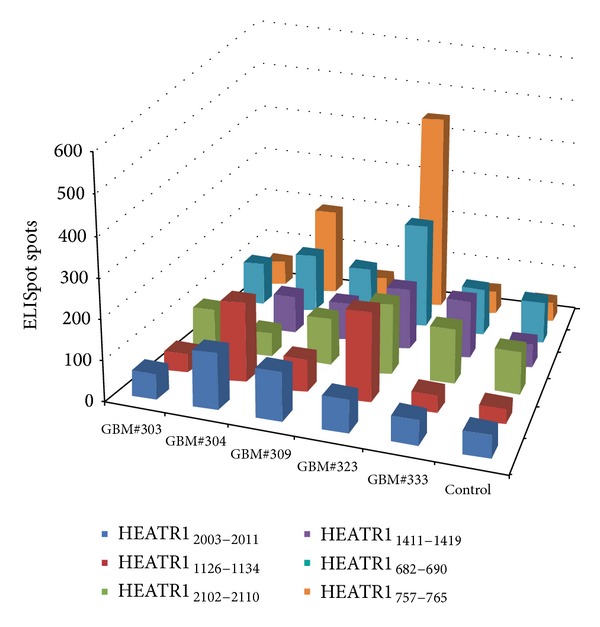
Single epitope peptide derived from the HEATR1 induces the IFN-*γ* response using ELISpot assay. PBMCs were extracted from 5 patients with HLA-A2+ GBM and 1 controlled patient with HLA-A∗02+ benign tumor. IFN-*γ* formingspots were calculated per 1 × 10^6^ PBMC.

**Figure 5:**
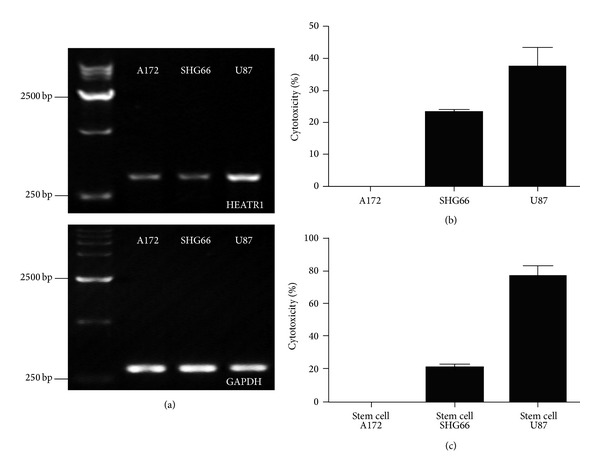
HEATR1-specific-peptide CTLs kill HLA-A∗02+ gliomas that express HEATR1. (a) RNA was isolated from three GBM cell lines and mRNA expression of HEATR1 was investigated by RT-PCR. (b) The values shown represent the mean ± SD of triplicate assays from PBMCs of patient number 323. U87, SHG66, and A172 were loaded with or without peptides and used as target cells in a LDH-release assay. The results showed that 6-peptides-stimulated PBMCs significantly lysed U87 and SHG66 target cells expressing both HEATR1 and HLA-A∗02 but not A172 cells that do not express HLA-A∗02 at an E/T ratio of 10 : 1. (c) Six-peptides-stimulated PBMCs from patient number 323 also significantly lysed the U87 and SHG66 GSCs at an E/T ratio of 10 : 1. Statistical differences between two groups were evaluated by the unpaired Student's *t*-test.

Furthermore, we investigated which individual HEATR1-derived peptide could induce the CTL responses. PBMCs from HLA-A∗02+ patients, five patients with GBM and one control patient with a benign tumor, were stimulated with individual peptide. As shown in Figure 4, HEATR1_757–765_ had the highest ELISpot response, indicating that it is the most immunogenic* in vivo*. In addition, the ELISpot responses induced by HEATR1_682–690_ and HEATR1_1126–1134_ were higher than the others (Figure 5). These data indicate that these three peptides possess the ability to induce CTLs* in vivo*.

### 3.5. HEATR1-Specific CTLs Lyse GBM Cells and GSCs

Finally, we evaluated the ability of HEATR1-specific CTLs to lyse GBM cell lines endogenously expressing HEATR1* in vitro*; all three GBM cell lines (U87, SHG66, and A172) are capable of expressing endogenous HEATR1 with the highest expression in U87 cell lines (Figure 5(a)). The cytotoxic activity of patients' PBMCs (effector cells) was evaluated using an LDH-release assay. PBMCs of patient 323 (positive ELISpot response with HLA-A∗02+; Table 1) were incubated with three GBM cell lines (U87, SHG66, and A172) as target cells, respectively. The results showed that peptide-stimulated PBMCs could lyse 37.4% of U87 and 23.1% of SHG66 target cells expressing both HEATR1 and HLA-A∗02 at an E : T ratio of 10 : 1 but not A172 cells that are HLA-A∗02-negative (Figure 5(b)). We further evaluated whether CTLs recognizing the HEATR1 peptides could kill A2B5+ GSCs. PBMCs from patient 323 demonstrated the ability to kill 76.8% of A2B5+ U87 GSCs and 20.4% of A2B5+ SHG66 GSCs at an E : T ratio of 10 : 1 (Figure 5(c)). These data suggest that HEATR1-specific CTLs are effective to lyse target cells endogenously expressing HEATR1; the cytotoxicity is associated with the expression level of endogenous HEATR1.

## 4. Discussion

To our knowledge, we reported first here that HEATR1 was especially overexpressed in GBM cells and A2B5+GBM cells. T-cell epitopes derived from HEATR1 could significantly induce the CTL response* in vivo *and these CTLs were able to lyse both GBM cells and GSCs. These results indicate that HEATR1 has great potential for the development of glioma immunotherapy.

The HEATR1 gene is a multiple spliced 7-kb gene that encodes bap28, a protein involved in nucleolar processing of pre-18S ribosomal RNA and ribosome biosynthesis. In the zebrafish central nervous system, bap28 is required for cell survival through its role in rRNA synthesis and processing, and its mutation leads to abnormalities in the brain starting at mid-somitogenesis stages [33]. A recent study indicated that HEATR1 is an ideal minor histocompatibility antigen that is expressed by leukemia stem cells [20, 34]. Moreover, HEATR1 expression detected using TaqMan PCR was higher in testicular and ovarian tissues than in liver, colon, small intestine, lung, brain, and heart tissues [20]. In the meantime, the novel polymorphic minor histocompatibility antigen encoded by the HEATR1 gene could be recognized by one of the CTL clones. In GBM, we first confirmed that HEATR1 expression was significantly higher in most of the GBM samples than in control brain tissues. Although HEATR1 overexpression was not detected in a few cases of GBM, it might contribute to the vast genetic aberrations and their heterogeneity of GBM or GBM samples from the tumor-surrounding tissues. Furthermore, HEATR1 was overexpressed in A2B5+ GSC cells compared to A2B5−tumor cells.

To date, T-cell epitopes derived from several glioma-associated antigens have been shown to elicit T-cell responses against gliomas of several genes, including SART-1 and -3, interleukin-13 receptor a2 chain, ARF4L, GALT3, AIM-2, EphA2, EGFRvIII, HER-2, gp100, MAGE-1, glioma big potassium (gBK),* TRP-2*,* SOX2*,* SOX11*,* SOX6*, and 3′  *β*-hydroxysteroid dehydrogenase type 7 gene [12, 24, 35–50]. Dutoit et al. recently reported that the peptidomes from* ex vivo* GBM samples, which consisted of 10 glioblastoma-associated antigen epitopes, induced specific tumor cell lysis by patients' CD8+ T-cells* in vitro* and* in vivo *[51]. Geet al. confirmed that gBK channel-specific peptides could induce HLA-A∗02-restricted human CD8+ CTLs that killed gBK+ tumor cells [50]. In our study, we confirmed that peptide epitopes derived from HEATR1 could significantly induce the CTL response of killing both GBM cells and A2B5+ GBM progenitor cells.

The CTL response in this study occurred in a non-HLA-A∗02-dependent manner. We found that HEATR1_1126–1134_ and HEATR1_1411–1419_ were also predicted to bind in the HLA-A∗03, HLA-B∗08, HLA-B∗38:01, and HLA-B∗40 regions using the epitope prediction system of SYFPEITHI analysis database (http://www.syfpeithi.de/bin/MHCServer.dll/EpitopePrediction.htm). Furthermore, positions 2 and 9 anchor peptides in the HLA-A∗02-peptide-binding groove are critical for optimal binding to HLA-A∗02. Positions 2 and 9 anchor peptides of those six peptides derived from HEATR1 were LL, LI, and MV, respectively (Table 2). More than 120 predicted peptides in non-HLA-A∗02 MHC class I (especially in HLA-B∗08) were found, where the 2nd and 9th positions were LL, LI, and MV. In addition, the HEATR1 region also was predicted to bind at least 1000 different 15-mers to the HLA-DR regions in the SYFPEITHI analysis database that could stimulate various CD4+ T-cells. Thus, six HEATR1 peptides in this study could cross-bind to the MHC class I or MHC class II region and potentially can be used to treat patients with GBM.

Several studies have used brain tumor stem-like initiating cells or cancer stem-like cells as sources of antigens for DC vaccination against human GBM with the achievement of CSC targeting and enhancing antitumor immunity [11–14]. GBM-associated tumor antigens including EGFR, HER2, TRP2, MRP3, AIM2, and SOX2 were twofold to >200-fold higher in CSCs than those in adherent cells [11]. Brown et al. reported that IL13-zetakine^+^ CTLs were capable of efficient recognition and killing of both IL13R*α*2^pos^ GSCs and IL13R*α*2^pos^ differentiated cells* in vitro* and* in vivo* [15]. Sampson et al. reported that EGFRvIII is expressed in GSC lines and EGFRvIII chimeric antigen receptors-engineered T-cells effectively target these lines [52]. However, the number of GSC-associated proteins' peptide epitopes known to elicit T-cell responses is rather limited, and sox6 is the first protein expressed in glioma stem cells whose peptides are potentially immunogenic in patients with HLA-A∗24 or -A∗02 positive glioma [12]. A2B5 is considered a marker for glioma progenitor cells and A2B5+ cells from human GBM have cancer stem cell properties that are crucial to GBM initiation and maintenance [17, 18]. In our study, we confirmed that HEATR1-derived peptide epitopes could significantly induce the CTL response and then lyse cells from the GBMs and the GSCs, which should be considered a promising strategy for effective T-cell-based immunotherapy for patients with GBM.

HEATR1 expression in normal brain tissues was very low, unlike* ARF4L* and* GALT3*, which were markedly expressed in various normal tissues [43, 44]. Interestingly, HEATR1-specific CTLs are only detectable in PBMC derived from patients with malignant gliomas but not in PBMC from healthy donors. Two reasons might account for this discrepancy. First, the induction of HEATR1-specific CTLs may require higher level of HEATR1 expression. As shown in Figure 1, HEATR1 expressions are significantly higher in tumors than in normal tissues. In other words, it is possible that the epitopes expressed in normal tissues are below the threshold level to stimulate T-cell responses [53]. Second, tumor induced microinflammation may result in the increase of permeability of blood-brain barrier and thereby help CTL to access and recognize the presented HEATR1-derived peptide on tumor cells. Furthermore, although the MAGE-1, MAGE-3, Melan-A, gp100, tyrosinase, HER-2, and NY-ESO-1 are expressed in normal testicular, retinal, and/or brain tissues, no autoimmune responses have been elicited in the clinical trials or animal experiments of cancer vaccines [54–58]. Of course, our results require further* in vivo* experiments to confirm the safety and effectiveness of those HEATR1-derived epitope peptides as future immunotherapy for patients with GBM.

## 5. Conclusion

In this study, we demonstrated the selective overexpression of HEATR1 in A2B5+ GBM cells, whose epitopes could induce specific CTL responses targeting GBM cells and GSCs, suggesting that immunotherapy selectively targeting GSCs could be a novel effective strategy to treat patients with malignant glioma. Combined with other therapeutic avenues, epitope-based GSC-targeting immunotherapy may represent a new promising paradigm for the treatment of patients with GBM [59, 60]. Moreover, those novel CTL epitopes may serve as an attractive component of personalized peptide-based vaccines in the treatment of GBM.

## Supplementary Material

Supplementary Figure 1: Double immunofluorescence staining of A2B5 and CD133.
Supplementary Figure 2: Double immunofluorescence staining of A2B5 and nestin.
Supplementary Figure 3: Double immunofluorescence staining of A2B5 and vimentin.
Supplementary Table 1: HLA subtype of patients with positive ELISpot response.

## Figures and Tables

**Table 1:** Clinical characteristics of healthy donors and patients.

Number	Gender	Years	Tumor location	Pathology	Grade	ELISpot response	HLA-A2
198	M	54		Healthy		Negative	Yes
203	M	55		Healthy		Negative	Yes
205	F	56		Healthy		Negative	Yes
209	F	45		Healthy		Negative	Yes
219	M	22		Healthy		Negative	No
306	M	31		Healthy		Negative	No

215	F	48	Right frontal	Meningioma	0	Negative	Yes
216	F	62	Cerebellum	Hemangioblastoma	0	Negative	No
255	F	59	Left frontal	Meningioma	0	Negative	Yes
226	F	49	Sellar region	Pituitary adenoma	0	Negative	Yes
261	M	21	Sellar region	Pituitary adenoma	0	Negative	No

122	M	45	Right temporal^¶^	Astrocytoma	2	Negative	No
135	F	37	Right temporal^¶^	Astrocytoma	2	Negative	Yes
217	M	48	Right frontal^¶^	Astrocytoma	2	Negative	No
246	F	30	Left parietal	Astrocytoma	2	Negative	Yes
252	M	50	Right temporal	Oligodendroglioma	2	Negative	No
264	M	45	Right frontal-callosum	Astrocytoma	2	Negative	Yes
262	M	40	Left temporal	Oligodendroglioma	2	Negative	Yes

218	M	58	Right temporal	AA	3	Negative	Yes
238	F	56	Right callosal convolution^¶^	AO	3	Negative	No
254	F	33	Right frontal	AOA	3	Negative	Yes
256	F	46	Right temporal-basal ganglia	AA	3	Negative	No
259	F	58	Right parietal	AO	3	Negative	No
265	M	56	Right frontal-parietal^¶^	AOA	3	Negative	No
127	F	42	Left frontal	AA	3	Positive	No
140	M	45	Left temporal	AA	3	Positive	A0201/A1101
156	M	48	Left occipital	AE	3	Positive	A0203/A3001
239	M	16	Left temporal	AA	3	Positive	A0207/A1102

129	F	38	Left occipital	GBM	4	Negative	Yes
133	F	48	Left temporal	GBM	4	Negative	No
147	F	61	Left temporal	GBM	4	Negative	Yes
150	M	48	Right frontal-temporal	GBM	4	Negative	Yes
151	M	30	Right temporal	GBM	4	Negative	Yes
214	M	66	Right parietal-occipital	GBM	4	Negative	No
223	M	37	Left frontal-temporal^¶^	GBM	4	Negative	Yes
224	M	59	Left temporal-occipital	GBM	4	Negative	Yes
225	M	31	Left frontal^¶^	GBM	4	Negative	No
231	M	36	Left frontal	GBM	4	Negative	No
241	M	58	Right frontal^¶^	GBM	4	Negative	Yes
253	M	54	Left frontal	GBM	4	Negative	No
132	M	68	Right temporal	GBM	4	Positive	A0201/A0203
141	F	12	Right parietal-occipital^¶^	GBM	4	Positive	No
220	M	47	Left temporal	GBM	4	Positive	A0201/A3303
221	F	39	Right temporal	GBM	4	Positive	No

¶: recurrence; F: female; M: male; GBM: glioblastoma multiforme; AA: anaplastic astrocytoma; AO: anaplastic oligodendroglioma; AOA: anaplastic oligoastrocytoma; AE: anaplastic ependymoma.

**Table 2:** Binding score of HEATR1-derived peptides to HLA-A02 molecules.

Peptide	HLA molecule	Amino acid position	Subsequence residue listing	Score (estimate of half time of disassociation of a molecule containing this subsequence)
HEATR1_2003–2011_	A_0201	2003–2011	F**L**FDTQHF**I**	4004.119
HEATR1_1126–1134_^&^	A_0201	1126–1134	K**L**LRMLFD**L**	3690.419
HEATR1_2102–2110_	A_0201	2102–2110	L**L**PESIPF**L**	1883.533
HEATR1_1411–1419_^¶^	A_0201	1411–1419	F**L**WILLIL**L**	1875.918
HEATR1_682–670_	A_0201	682–670	K**M**VEDLIS**V**	1657.907
HEATR1_757–765_	A_0201	757–765	L**M**LDRGIP**V**	1295.433

^&^HEATR1_1126–1134_ was also predicted to bind to HLA-03 and HLA-B08.

^¶^HEATR1_1411–1419_ was also predicted to bind to HLA-B08, HLA-B40, and HLA-B3801.

## Abbreviations

GBM: Glioblastoma
GSCs: Gliomas stem-like cells
CSCs: Cancer stem-like cells
FACS: Fluorescence-activated cell sorting
DC: Dendritic cell
HEATR1: HEAT repeat containing 1
PBMCs: Peripheral blood mononuclear cells
CTL: Cytotoxic T lymphocyte
Q-RT-PCR: Quantitative reverse transcription-polymerase chain reaction
HLA: Human leukocyte antigen
IFN: Interferon.
