# Supplementary material for: Glioma-Associated Antigen HEATR1 Induces Functional Cytotoxic T Lymphocytes in Patients with Glioma
Source: J Immunol Res. 2014 Jul 9;2014:131494. doi: 10.1155/2014/131494 (PMC4121097; doi:10.1155/2014/131494)
Supplement: Supplementary file 1 — Supplementary Figure 1: Double immunofluorescence staining of A2B5 and CD133. Supplementary Figure 2: Double immunofluorescence staining of A2B5 and nestin. Supplementary Figure 3: Double immunofluorescence staining of A2B5 and vimentin. Supplementary Table 1: HLA subtype of patients with positive ELISpot response. [file 131494.f1.doc]

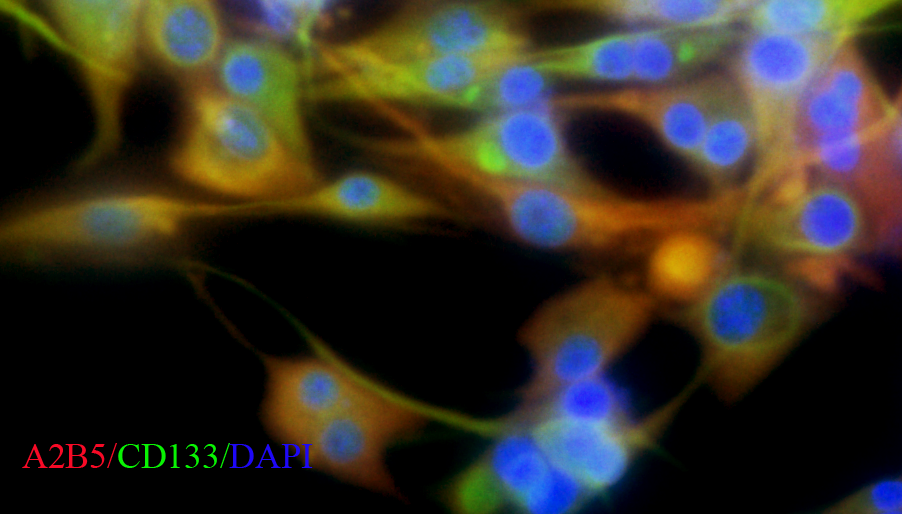


**Supplementary Figure 1.** Double immunofluorescence staining of A2B5 and CD133.


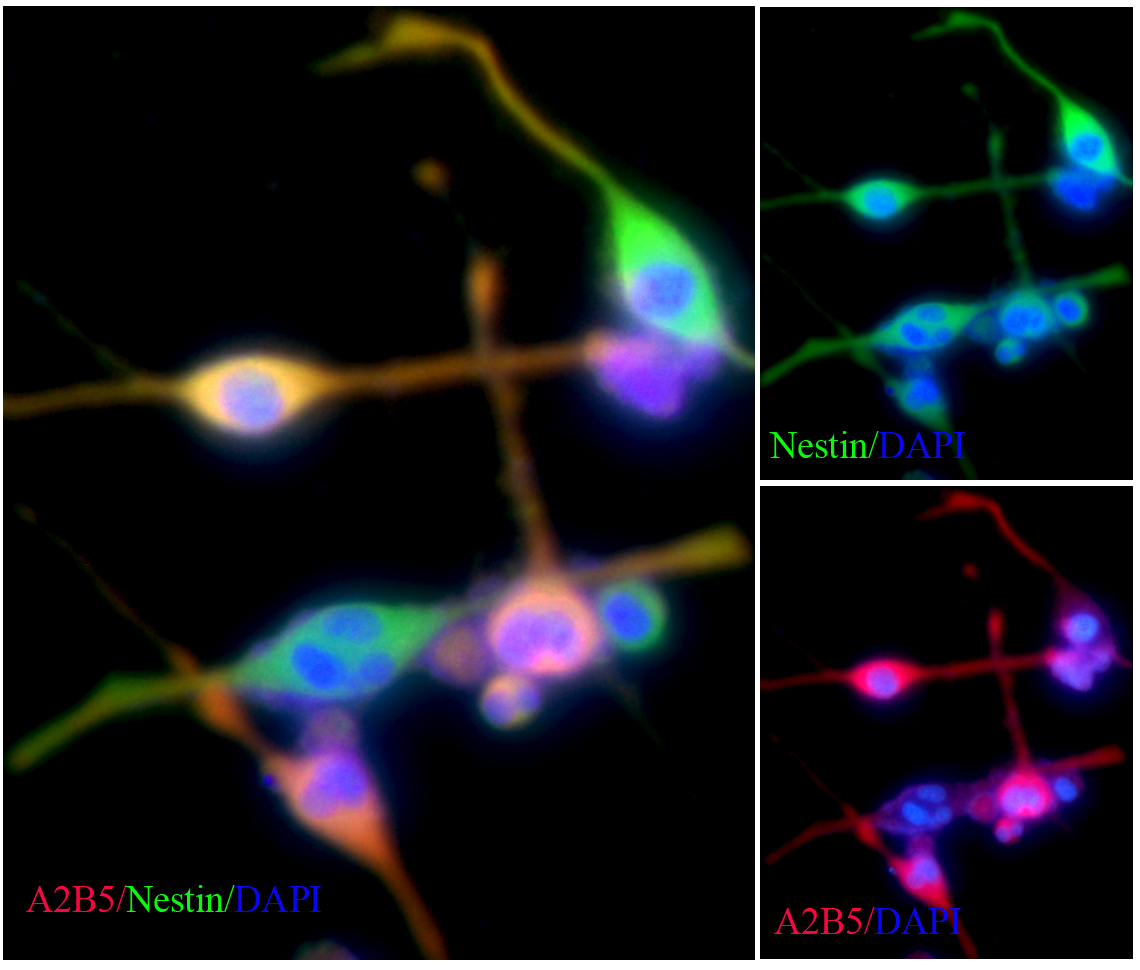


**Supplementary Figure 2.** Double immunofluorescence staining of A2B5 and nestin.


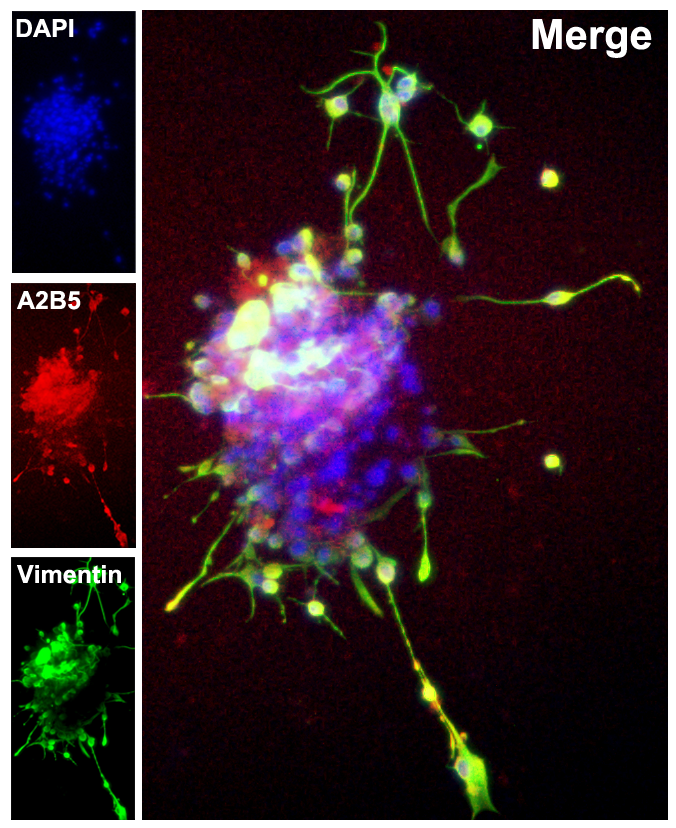


**Supplementary Figure 3.** Double immunofluorescence staining of A2B5 and vimentin.
